# Exposure Time Control Method for Higher Intermediate Frequency in Optical Heterodyne Imaging and Its Application to Electric-Field Imaging Based on Electro-Optic Effect

**DOI:** 10.3390/s24041249

**Published:** 2024-02-15

**Authors:** Kiyotaka Sasagawa, Ryoma Okada, Yoshihiro Akamatsu, Maya Mizuno, Hironari Takehara, Makito Haruta, Hiroyuki Tashiro, Jun Ohta

**Affiliations:** 1 Division of Materials Science, Graduate School of Science and Technology, Nara Institute of Science and Technology, 8916-5 Takayama, Ikoma, Nara, 630-0192 Japan; okada.ryoma.on9@ms.naist.jp (R.O.); akamatsu.yoshihiro.as8@ms.naist.jp (Y.A.); t-hironari@ms.naist.jp (H.T.); m-haruta@photon.chitose.ac.jp (M.H.); tashiro.hiroyuki.289@m.kyushu-u.ac.jp (H.T.); ohta@ms.naist.jp (J.O.); 2 Radio Research Institute, National Institute of Information and Communications Technology, 4-2-1, Nukui-kitamachi, Koganei, Tokyo 184-8795, Japan; mmizuno@nict.go.jp; 3 Department of Opto-Electronic System Engineering, Chitose Institute of Science and Technology, 758-65 Bibi, Chitose, Hokkaido 066-8655, Japan; 4 Department of Health Sciences, Faculty of Medical Sciences, Kyushu University, 3-1-1, Maidashi, Higashi-ku, Fukuoka, 812-8582, Japan

**Keywords:** electric-field imaging, electro-optic effect, equivalent time sampling, image sensor, optical heterodyne

## Abstract

We propose and demonstrate a method for equivalent time sampling using image sensors to selectively detect only the target frequency. Shortening the exposure time of the image sensor and using equivalent time sampling allows for the detection of frequency components that are higher than the frame rate. However, the imaging system in our previous work was also sensitive to the frequency component at 1/4 of the frame rate. In this study, we control the phase relationship between the exposure time and observed signal by inserting an additional interval once every four frames to detect the target frequency selectively. With this technique, we conducted electric field imaging based on the electro-optic effect under high noise conditions in the low-frequency band to which the conventional method is sensitive. The results demonstrated that the proposed method improved the signal-to-noise ratio.

## 1. Introduction

Image sensors can acquire information from multiple points in parallel, thereby enabling the simultaneous acquisition of various distributions. Its most common application is in the acquisition of photographs and moving images. Signal processing of the light information from individual pixels helps acquire numerous information, rather than a simple light intensity distribution [1]. Also, signal processing from a large number of frame data can be used to extract intensity and phase information from the temporal changes in the pixel signals.

Some methods for achieving phase detection involve signal processing within the pixel to handle relatively high frequencies [2,3,4,5]. However, implementing this may be challenging because of the complexity of the pixel structure. Also, the response frequency may be insufficient in our targeted high-frequency electric-field imaging.

The optical heterodyne method can convert the frequency into an intermediate frequency band when measuring high-frequency signals using a relatively slow photodetector. One application involves high-frequency electric-field imaging using electro-optic (EO) crystals [6,7,8,9,10,11,12,13,14,15,16] that change their birefringence upon applying an electric field. When light is irradiated onto an EO crystal, the polarization state is modulated as a result of changes in birefringence, which can be analyzed to obtain electric field information.

The advantage of this electric field measurement method is that the EO crystal probe is metal-free and causes minimal disturbance to the electromagnetic field to be observed [11,12,14,15,16,17,18,19]. Furthermore, when the probe is combined with an image sensor, multiple points can be measured at once, and the electric field distribution can be visualized quickly [20]. EO crystals respond to high frequencies on the order of terahertz [21,22,23,24,25,26,27,28], but they cannot be directly observed using slow optical detectors such as image sensors. Therefore, frequency conversion using optical heterodyne detection is necessary.

**Figure 1:**
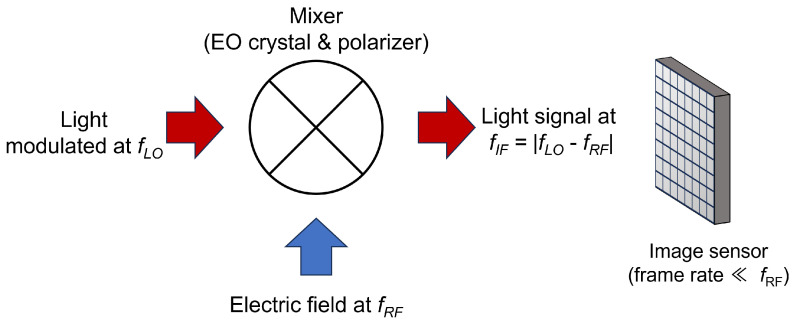
Schematic of frequency conversion by optical heterodyne.

A schematic of the optical heterodyne method is shown in Figure 1. Here, the combination of an EO crystal and detector acts as a kind of mixer. The EO crystal is irradiated with intensity-modulated light at a local oscillator frequency $f_{LO}$, slightly different from the frequency $f_{RF}$ signal of the observation target. The light transmitted through the crystal and the polarizer as an optical polarization analyzer contains an intermediate-frequency component, $f_{IF}=\left|f_{LO}-f_{RF}\right|$. The intensity and phase information of the high-frequency signal can be obtained by setting the intermediate frequency below the frame rate of the image sensor. In previous works, we have demonstrated obtaining the intensity and phase distributions of a high-frequency electric field using our originally designed polarization image sensor [29]. The role of the image sensor in our system is to detect, in parallel, signals containing high-frequency electric field information that are down-converted to intermediate frequencies by the optical heterodyne method. However, because of the limitations of the image sensor we used [30], the frame rate was less than 100 Hz. Therefore, the intermediate frequency had to be set very low. In this condition, due to noises, such as those from commercial power sources or mechanical vibrations, the signal-to-noise ratio (SNR) is easily reduced. In this study, we propose a method for detecting a higher intermediate frequency component while maintaining the frame rate, and demonstrate that SNR can be improved when affected by noise in the low frequency band.

The rest of this paper is organized as follows. Section 2 explains the proposed equivalent time sampling method. Section 3 describes the image sensor and experimental EO imaging system that we used to test the proposed method. In Section 4, we present the high-frequency field imaging results and demonstrate the effectiveness of the proposed method. In Section 5, we discuss the effectiveness of the SNR improvement based on the experimental results. Finally, in Section 6, we conclude with a discussion of the implications of this work.

## 2. Proposed Method

The essential detection condition for combining the optical heterodyne method with an image sensor is that the intermediate frequency is 1/4 of the frame rate. Because four frames correspond to one cycle of the intermediate frequency, the signal processing for phase detection is simplified [31]. This is because the in-phase and quadrature components required for phase detection have a phase difference of 1/4 cycle from each other. If an image with frame number *n* is represented by $I_{n}$, then the in-phase and quadrature components are calculated as follows:

(1) $$\begin{array}{cc} S_{inphase} & =\sum_{n=0}^{N/4}\left(I_{4n+1}+I_{4n+2}-I_{4n+3}-I_{4n+4}\right) \end{array}$$

(2) $$\begin{array}{cc} S_{quad} & =\sum_{n=0}^{N/4}\left(I_{4n+1}-I_{4n+2}-I_{4n+3}+I_{4n+4}\right) \end{array}$$

where *N* is the number of total frames used in the calculation. The intensity and phase of the signal is provided by $S_{inphase}^{2}+S_{quad}^{2}$ and the arctangent of $S_{quad}/S_{inphase}$, respectively.

The advantage of calculating under these conditions is that it involves adding and subtracting frames, making them highly compatible with digital signal processing. Imaging systems that use this feature for real-time signal processing have been reported [31].

**Figure 2:**
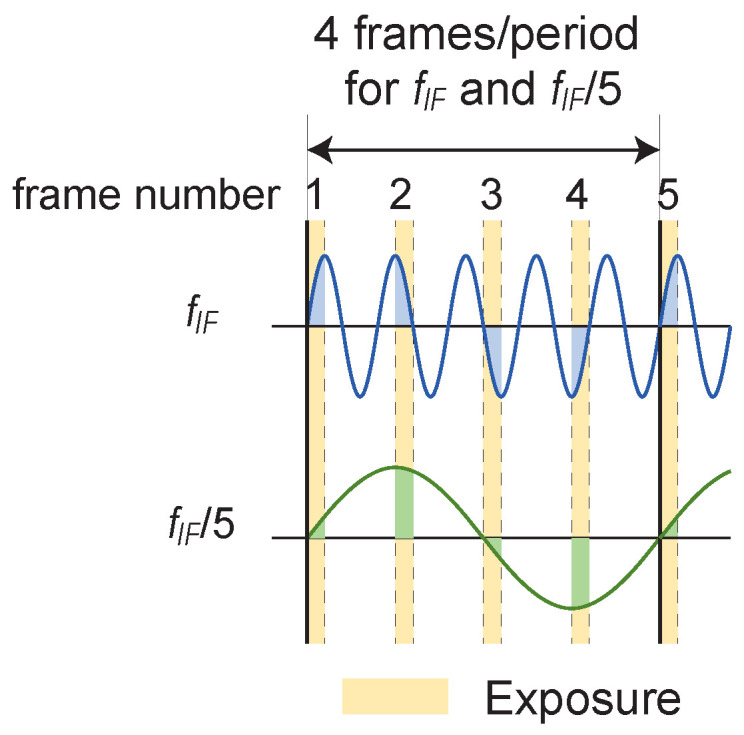
Relationship between signal and exposure time in the equivalent time sampling.

The frame rate improvement is effective at detecting high-frequency components. However, image sensors with a frame rate of several kfps, for example, are special and difficult to achieve, particularly when the number of pixels is large. However, one can control the exposure time even with an image sensor drive system with a relatively low frame rate using equivalent time sampling. To extend the aforementioned signal processing to a set of four frames, we increase the intermediate frequency to (4M+1)/4 times the frame rate (M=1,2,3,⋯). The relationship between the frame and intermediate frequency signal is shown in Figure 2.

When the exposure time is $T_{exp}$ and time per frame is $T_{frame}$, their relationship is provided by

(3) $$\begin{array}{c} T_{exp}=\frac{1}{4M+1}T_{frame}. \end{array}$$

Increasing *M* allows for higher frequencies to be detected. However, shortening the exposure time while maintaining a constant frame rate increases the amount of irradiated light that is not detected. In other words, the utilization efficiency of the irradiated light is reduced. This study provides a proof-of-principle demonstration for *M* = 1; i.e., $T_{exp}=\left(1/5\right)T_{frame}$.

Setting the exposure time to a 1/4 cycle relative to the frequency component $f_{IF}$ of the observation target and setting the frame rate to $f_{IF}/5$ allows for extracting frequency components higher than the frame rate. However, the $f_{frame}/4$ component, which is 1/4 of the frame rate, is also detected under this condition. This occurs because the time of one cycle is the same for the frequency $f_{IF}$, which is the intermediate-frequency component, and for the $f_{IF}/5\left(=f_{frame}/4\right)$ component, as shown in the lower part of Figure 2. They cannot be separated using the phase detection process mentioned in the previous section.

The purpose of introducing equivalent-time sampling in this study is to avoid noise in the lower-frequency range. However, if the frequency component in $f_{IF}/5\left(=f_{frame}/4\right)$ is detected, SNR is not improved due to the noise in this frequency band.

One of the ways to solve this problem is to make one period of the signal used for a longer phase detection. In this study, the interval time is increased by 1/4 cycle every four frames, i.e., the frame start time is delayed once every four frames.

By adding an additional interval time, the phase relationship with the intermediate-frequency component changes every four frames, that is, every frame set. However, phase detection can still be performed by a simple add/subtract process. We perform phase detection by selecting an appropriate sign and processing four sets of 16 frames to detect the desired frequency components.

In this case, phase detection Equations (1) and (2) can be rewritten as follows:

(4) $$\begin{array}{cc} S_{inphase}^{\prime } & =\sum_{n=0}^{N/16-1}(I_{16n+1}+I_{16n+2}-I_{16n+3}-I_{16n+4}\\ \; & \;+I_{16n+5}-I_{16n+6}-I_{16n+7}+I_{16n+8}\\ \; & \;-I_{16n+9}-I_{16n+10}+I_{16n+11}+I_{16n+12}\\ \; & \;-I_{16n+13}+I_{16n+14}+I_{16n+15}-I_{16n+16}) \end{array}$$

(5) $$\begin{array}{cc} S_{quad}^{\prime } & =\sum_{n=0}^{N/16-1}(I_{16n+1}-I_{16n+2}-I_{16n+3}+I_{16n+4}\\ \; & \;+I_{16n+5}-I_{16n+6}-I_{16n+7}+I_{16n+8}\\ \; & \;-I_{16n+9}+I_{16n+10}+I_{16n+11}-I_{16n+12}\\ \; & \;+I_{16n+13}+I_{16n+14}-I_{16n+15}-I_{16n+16}) \end{array}$$

For a component with a frequency of $f_{frame}/4$, the phase shift differs from that of the $f_{IF}$ component. Because the frequency is 1/5, one period has 80 (=5 × 16) frames. Instead of repeatedly sampling four portions of the one cycle, as shown in Figure 2, all portions of the cycle are sampled in 80 frames. In other words, the $f_{IF}/5$ component is canceled by integration, and the frequency of interest $f_{IF}$ can be selectively detected. The effective frame rate is slightly reduced because of the delay once every four frames, and it is provided by the following equation:

(6) $$\begin{array}{c} f_{frame}^{\prime }=\frac{4f_{frame}}{4+\frac{1}{4N+1}} \end{array}$$

As in the simple time equivalent sampling method, the intensity is the sum of the squares of the in-phase and quadrature components, and the phase is the arc tangent to the ratio of these components, respectively.

**Figure 3:**
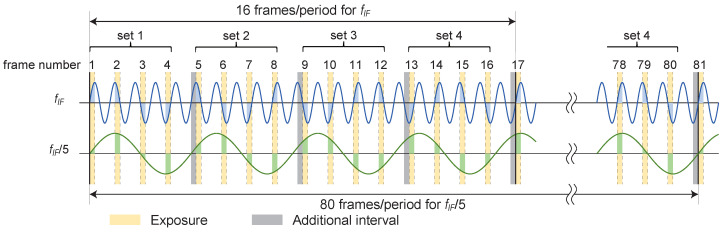
Relationship between signal and exposure time for equivalent time sampling in the proposed method. We insert an interval corresponding to 1/4 cycle of the signal to be observed once every four frames.

In the above description, an interval of 1/4 cycle is added once every four frames. The purpose was to shift the phase of the $f_{IF}/5$ component in Figure 3 by one set of exposure times so that the result of the integration cancels out. Thus, for a natural number $N_{S}\left(=1,2,3,\cdots \right)$, this condition is satisfied by inserting an additional interval once every $4N_{S}$ or $4N_{S}+2$ frames. In other words, the frequency of inserting additional intervals can be lower than in the above example. This results in a lower rate of frame rate reduction. On the other hand, more frames are required to accurately cancel the $f_{IF}/5$ component.

## 3. Experimental Setup

**Figure 4:**
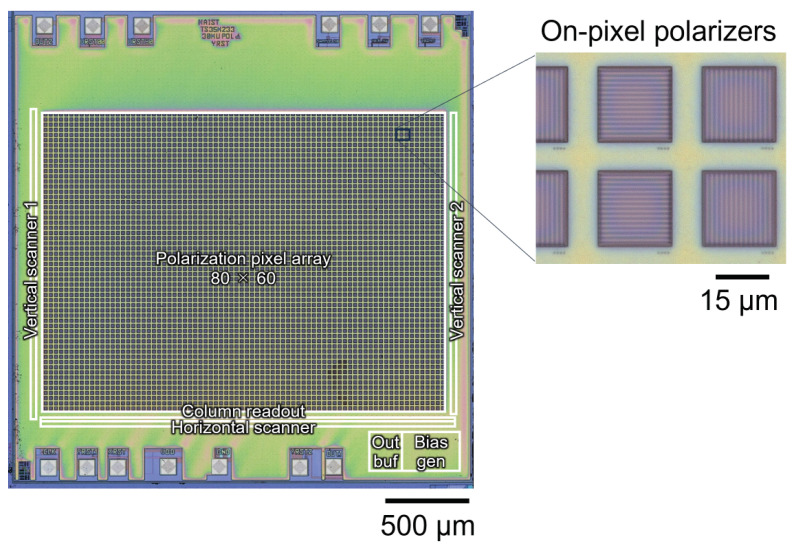
Micrograph of the image sensor with on-pixel polarizers. Inset shows a magnified view of the pixels.

We conducted experiments using our field-imaging system to demonstrate the proposed method. The chip used in this study is the same as in a previous study [29,30]. However, the design was modified to adjust the exposure time. The chip micrograph of the fabricated sensor is shown in Figure 4. This image sensor, fabricated with a 0.35-μm 2-poly 4-metal standard CMOS process, was used as the prototype. The specifications are listed in Table 1. The number of pixels was 80 × 60, and a polarizer was fabricated on each pixel using the wiring layers of the CMOS process [32]. Polarization image sensors usually consist of a set of pixels with polarizers at different angles of $45^{\circ }$ increments [33,34,35,36,37,38,39,40,41,42,43]. Our polarization image sensor, on the other hand, used a set of two pairs of polarization pixels with polarizer angles orthogonal to each other. This configuration was used to detect slight changes in polarization.

For polarizers, two metal gratings were stacked to achieve a high extinction ratio [44]. The extinction ratio of the polarization pixel in this work was approximately 3.1 at a wavelength of 780 nm. This value was much lower than that of state-of-the-art on-pixel polarizers [34,39,41,42]. However, in our proposed dual polarizer setup, a high polarization change sensitivity could be achieved with relatively low extinction ratio pixels [29,30].

In this image sensor, pixel information is read using a rolling shutter system. Rolling shutters have the advantage of simplifying the pixel structure, resulting in a difference in the exposure time for each row. In the case of phase detection, as in this study, the phase signal obtained is shifted as the row advances. However, this is not a major problem because the exposure time difference can easily compensate for phase differences in each row of the image sensor.

**Figure 5:**
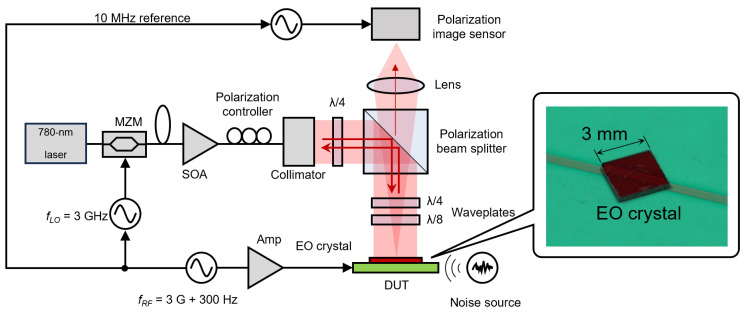
Experimental setup for the electric-field imaging. Red arrows show the directions of light.

Figure 5 shows the optical system used for electric field imaging. This is almost the same as that in our previous study [45]. We used a single-wavelength laser diode (780 nm) as the light source. Electric-field measurements based on EO effects are possible at other wavelengths, for example, the 1.55-µm band is often used for single-point measurements, but it is difficult to fabricate high-quality image sensors outside of the Si process. On the other hand, the 780 nm band is detectable by Si image sensors. Also, semiconductor optical amplifiers (SOA) are available. As EO crystals, ZnTe, which has a Zinc Blende structure, has no birefringence in the absence of an applied electric field, and has a large EO coefficient, is often used [21,22,24,46]. However, ${\mathrm{LiNbO}}_{3}$, which is used in optical modulators and of which high-quality crystals are readily available, can also be used [25,26,28]. Also, high-performance optical modulators are available in this wavelength band. Light from a continuous wave laser source is modulated with a Mach–Zehnder-type intensity modulator at a frequency corresponding to $f_{LO}$ and irradiates the EO crystal through a polarization beam splitter (PBS) and waveplates.

The device under test (DUT) was a microstrip line fabricated on a substrate with a high dielectric constant (RT/duroid 6010.2LM) of approximately 10. It was designed so that the characteristic impedance of the line was approximately 50 Ω.

In this study, we used a 0.3 mm thick (100)-ZnTe crystal with a resistivity of > $10^{3}\;\Omega \cdot$cm (JX Metals, Tokyo, Japan) as the EO probe. This crystal is sensitive to electric fields perpendicular to its surface. In this study, antireflection and high-reflection coatings for the wavelength of 780 nm were deposited on the top and bottom surfaces of the crystal, respectively. The output power from SOA was set to approximately 6 and 30 mW for the normal and equivalent time sampling, respectively, to ensure similar pixel outputs. The experiments were conducted at room temperature.

The optical system used in this study has a dual polarizer configuration consisting of on-pixel polarizers and a PBS. PBS is a uniform polarizer, and it was positioned to have a cross-nicol relation with respect to the light reflected from the observed object. In this system, it acts as an optical element that reduces the amount of light and increases the degree of polarization rotation. On the other hand, the on-pixel polarizers convert the polarization change into the light intensity change. In general, image sensor pixels are small, and SNR is low due to the low light-receiving limit for pixel saturation. However, the dual polarizer configuration allows for detecting weak polarization changes with a high sensitivity. The details can be found in [30]. In this study, we set the frame rate of the image sensor to 240 Hz, the LO frequency to 3 GHz, and the high-frequency electric field to be observed to 3G + 300 Hz. The intermediate frequency was 300 Hz, 1.25 (=5/4) times the frame rate.

## 4. Results

**Figure 6:**
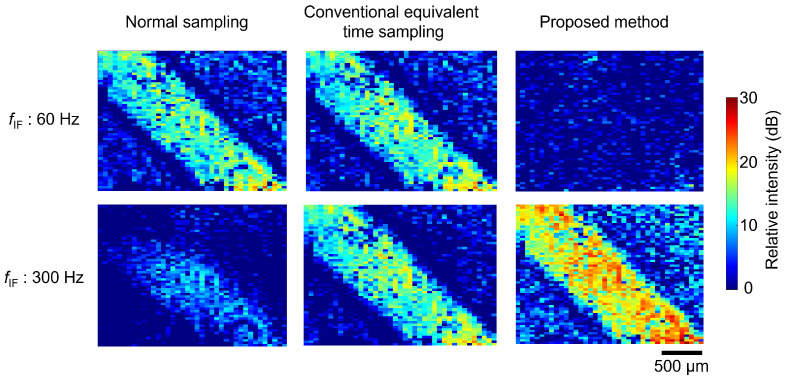
Electric-filed imaging results.

Figure 6 shows the observation results when $f_{IF}$ was set to 60 or 300 Hz. Each column shows the cases of normal sampling, simple equivalent time sampling, and the proposed method with an additional interval. The colors indicate the relative signal intensity with respect to the noise level when the high-frequency signal was not input into the DUT. The EO crystal used in this study, (100)-ZnTe, is sensitive to the electric fields applied perpendicular to the surface of the microstrip line. Therefore, the intensity was relatively high on the line when visualizing the distribution.

In normal sampling, the system was sensitive only when $f_{IF}$ was 60 Hz. In contrast, the result of the equivalent time sampling shows that the electric field was detectable at 300 Hz, which was 5/4 times the frame rate. However, the intensity distribution image was almost the same for $f_{IF}$ of 60 Hz, indicating that the sensitivity was comparable for both frequencies. This is because the 60-Hz component was also detected when performing the phase detection operation at 300 Hz, as discussed in Section 2. This means that if there was a significant noise at 60 Hz, it was detected, and SNR decreased. In contrast, in the proposed method, when $f_{IF}$ was 60 Hz, an electric field distribution was not observed in the image. As a result, higher relative signal intensities were obtained at 300 Hz compared to the other cases.

**Figure 7:**
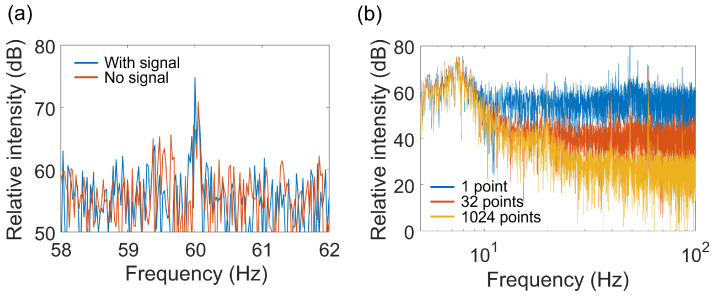
(**a**) Spectra near the mid-frequency at pixels on a microstrip line. (**b**) Noise spectra in the low-frequency range.

Figure 7a shows the fast Fourier transform (FFT) spectrum of the pixel output near the center of the image sensor image with normal sampling. This experimental result also shows that even when there was a strong noise at approximately 60 Hz, it caused a reduction in the SNR.

Figure 7b shows the results of a spectral comparison during pixel averaging, with no high-frequency signal input. The result shows that the white noise component was reduced by averaging.

## 5. Discussion

The experimental results show that the low-frequency component was successfully canceled by adding an interval corresponding to 1/4 cycle of the frequency of the observed object every four frames in the equivalent time sampling. Figure 6 shows that the proposed method provided a higher SNR than the conventional method. The noise source, which was the power supply line, provided noise at approximately 60 Hz. The proposed method had a reduced sensitivity in the 60 Hz frequency band, lowering the noise level. Because the signal level was similar to that in the other methods, the relative intensity with respect to the noise level, that is, SNR, was higher. Although we selected an intermediate frequency with a high noise for demonstration, the proposed method would improve SNR, even with a widely distributed noise in the low-frequency band. Equivalent time sampling has the disadvantage of reducing light utilization efficiency. In this study, it was compensated by introducing an optical amplifier. The best way to improve SNR by increasing the intermediate frequency is to increase the frame rate of the image sensor. However, the frame rate may be limited due to system constraints. The proposed method can increase the intermediate frequency and improve SNR without increasing the frame rate.

The intermediate frequency of 300 Hz used in this experiment is not necessarily high enough in some cases. For example, in single-point measurement systems, even higher frequencies, from tens of kHz to several MHz, are often used [10]. In this study, it was possible to increase the intermediate frequency to 1 kHz or higher by shortening the exposure time of the image sensor. However, shorter exposure times required higher light intensity. The optimal value depends on the tradeoffs between the magnitude of the external noise, the maximum output limit of the optical amplifier, and the increase in noise due to the higher amplification.

If the temporal or spatial resolution requirements are low, it is possible to improve SNR by integrating data from several frames or pixels. In Figure 7b, the white noise component is reduced by pixel averaging. Theoretically, when the number of pixels or frames used for phase detection is *N* times larger, the noise amplitude is reduced by a factor of $1/\sqrt{N}$. In Figure 7b, the rate of white-noise reduction with the increasing number of pixels used for averaging is consistent. The electric-field imaging results in Figure 6 show that the SNR for a single point on the microstrip line is approximately 19 dB. In contrast, when integrating data from the 1050 points on the line, SNR increases to approximately 52 dB, which agrees with the theory.

## 6. Conclusions

In this study, we proposed a method for detecting signals with a specific frequency higher than the frame rate using an equivalent time-sampling technique with image sensors, and applied it to an electric field imaging system using the EO effect. The proposed method has reduced sensitivity to low-frequency noise and improved the SNR in the detection system using an optical heterodyne.

The proposed method can be preprocessed in the digital domain using simple calculations, such as addition and subtraction. Therefore, the proposed method can also be applied to real-time imaging based on digital signal processing.

## Figures and Tables

**Table 1:** Specifications of the polarization CMOS image sensor.

Technology	0.35-µm 2-poly 4-metal standard CMOS
Operating voltage (V)	3.3
Pixel number	80 × 60
Pixel type	3-transistor active pixel sensor
Pixel Size (µ$m^{2}$)	30 × 30
Photodiode Size (µ$m^{2}$)	21 × 21
Photodiode type	Nwell-Psub
Polarizer	Line/Space = 0.7 µm/0.7 µm (2 layers)
Extinction ratio	3.1 @ 780 nm
Chip Area (µ$m^{2}$)	2700 (W) × 2645 (L)

## Data Availability

No new data were created or analyzed in this study. Data sharing is not applicable to this article.
